# Screening a Small Library of Xanthones for Antitumor Activity and Identification of a Hit Compound which Induces Apoptosis

**DOI:** 10.3390/molecules21010081

**Published:** 2016-01-13

**Authors:** João Barbosa, Raquel T. Lima, Diana Sousa, Ana Sara Gomes, Andreia Palmeira, Hugo Seca, Kantima Choosang, Pannee Pakkong, Hassan Bousbaa, Madalena M. Pinto, Emília Sousa, M. Helena Vasconcelos, Madalena Pedro

**Affiliations:** 1 CESPU, Instituto de Investigação e Formação Avançada em Ciências e Tecnologias da Saúde, IUCS—Instituto Universitário de Ciências da Saúde, Rua Central de Gandra 1317, Gandra 4585-116, Portugal; j.filipebarbosa@hotmail.com (J.B.); hbousbaa@gmail.com (H.B.); 2 i3S—Instituto de Investigação e Inovação em Saúde da Universidade do Porto, Rua Alfredo Allen 208, Porto 4200-135, Portugal; rlima@ipatimup.pt (R.T.L.); dsousa@ipatimup.pt (D.S.); hteixeira@ipatimup.pt (H.S.); 3 Cancer Drug Resistance Group, IPATIMUP—Institute of Molecular Pathology and Immunology of the University of Porto, Porto 4200-135, Portugal; 4 Department of Pathology and Oncology, FMUP—Faculty of Medicine of the University of Porto, Alameda Prof. Hernâni Monteiro, Porto 4200-319, Portugal; 5 Laboratory of Microbiology, Department of Biological Sciences, FFUP—Faculty of Pharmacy of the University of Porto, Rua de Jorge Viterbo Ferreira 228, Porto 4050-313, Portugal; 6 Laboratory of Organic and Pharmaceutical Chemistry, Department of Chemical Sciences, FFUP—Faculty of Pharmacy of the University of Porto, Rua de Jorge Viterbo Ferreira 228, Porto 4050-313, Portugal; anasarag4@gmail.com (A.S.G.); andreiapalmeira@gmail.com (A.P.); madalena@ff.up.pt (M.M.P.); esousa@ff.up.pt (E.S.); 7 UCIBIO/REQUIMTE, Laboratório de Microbiologia, Departamento de Ciências Biológicas, Faculdade de Farmácia, Universidade do Porto, Rua de Jorge Viterbo Ferreira 228, Porto 4050-313, Portugal; 8 Faculty of Medicinal Technology, Rangsit University, 52/347 Muang Ake, Phaholyothin Road, Lakhok, Pathumthani 10210, Thailand; kantima90@hotmail.com; 9 Applied Radiation and Isotopes Department, Faculty of Science, Kasetsart University, Jatujak, Bangkok 10930, Thailand; fscipnp@ku.ac.th; 10 CIIMAR/CIMAR—Centro Interdisciplinar de Investigação Marinha e Ambiental, Universidade do Porto, Rua dos Bragas 289, Porto 4050-123, Portugal

**Keywords:** antitumor activity screening, thioxanthones, *in vitro* cell growth assays, apoptosis

## Abstract

Our previous work has described a library of thioxanthones designed to have dual activity as P-glycoprotein modulators and antitumor agents. Some of these compounds had shown a significant cell growth inhibitory activity towards leukemia cell lines, without affecting the growth of non-tumor human fibroblasts. However, their effect in cell lines derived from solid tumors has not been previously studied. The present work aimed at: (i) screening this small series of compounds from an in-house library, for their *in vitro* cell growth inhibitory activity in human tumor cell lines derived from solid tumors; and (ii) initiate a study of the effect of the most potent compound on apoptosis. The tumor cell growth inhibitory effect of 27 compounds was first analysed in different human tumor cell lines, allowing the identification of a hit compound, **TXA1**. Its hydrochloride salt **TXA1·HCl** was then synthesized, to improve solubility and bioavailability. Both **TXA1** and **TXA1·HCl** inhibited the growth of MCF-7, NCI-H460, A375-C5, HeLa, 786-O, Caki-2 and AGS cell lines. The effect of **TXA1·HCl** in MCF-7 cells was found to be irreversible and was associated, at least in part, with an increase in cellular apoptosis.

## 1. Introduction

Thioxanthones are isosteric analogues of xanthones, consisting of S-heterocycles with a dibenzo-γ-thiopyrone scaffold. The first thioxanthone with promising therapeutic value, lucanthone (Miracil D), appeared in the decade of the 1940s as an antischistossomal agent [1,2]. Several studies on the biological activities of thioxanthones allowed their identification as anticancer agents, as well as the identification of their mechanisms of action [2]. In addition, it was found that treatment with some thioxanthones sensitized tumor cells to the effect of other chemotherapeutic agents, which enabled new chemotherapeutic approaches [2]. Regarding the mechanism of action of thioxanthones, lucanthone and its derivative hycanthone were found to be able to intercalate into DNA and to inhibit RNA synthesis, as well as the DNA topoisomerases I and II [3]. Nevertheless, although presenting similarity with other intercalating agents, their mutagenicity (mainly due to their methylene moiety directly bound to C-4) discouraged their use in cancer chemotherapy [2,4]. Other examples of thioxanthones with antitumor activity are SR233377 and SR271425 [5,6]. SR233377, a hycanthone derivative, is a second-generation aminated thioxanthone which presented selectivity for mouse solid tumors when compared to normal cells (using a disc diffusion *in vitro* assay) and was also confirmed to be active *in vivo* in tumors implanted in murine models [5]. Nevertheless, it was found to be hepatotoxic. This problem was latter overcome by the development of SR271425, a third-generation thioxanthone, which presented a broad-spectrum activity against solid tumors both *in vitro* and *in vivo* (in murine as well as in human xenograft tumor models) [6].

Although several thioxanthone derivatives have entered clinical trials as antitumor agents in the last decade, their toxicity has largely limited their clinical utility [2,5,6,7]. In order to circumvent this toxicity problem, which was associated with their pattern of substitution, and in order to improve their efficiency, a small library of new thioxanthone derivatives with potential as antitumor agents and simultaneously with P-glycoprotein (P-gp) inhibitory activity, was designed and recently obtained by some of us [4]. These derivatives presented an oxygenated function in C-4, instead of the methylene moiety associated with the toxicity exhibited by lucanthone. Even though some of these compounds were previously shown to have both antitumor (and anti P-gp) activity in leukemia cell lines, while not being toxic to non-tumor cells, their cell growth inhibitory activity in tumor cell lines derived from solid tumors had not been previously studied.

Therefore, the main aim of the present study was to screen this small series of thioxanthones regarding their cell growth inhibitory effect in a panel of human tumor cell lines derived from solid tumors and, in addition, to gain some insights into the mechanism of action of **TXA1·HCl**, the hydrosoluble hydrochloride derivative of the most potent compound, 1-{[2-(diethylamino)ethyl]-amino-4-propoxy-9*H*-thioxanthen-9-one (**TXA1**), in MCF-7 breast cancer cells.

## 2. Results and Discussion

### 2.1. Screening of a Small Library of Xanthones for Their Cell Growth Inhibitory Activity in Human Tumor Cell Lines Derived from Solid Tumors: Identification of ***TXA1*** as a Hit Compound

Previous studies carried out by some of us had shown that a library of thioxanthones **1**–**27** (Table 1) presented potent cell growth inhibitory effect in leukemia cell lines. In addition, these compounds had also been tested in MRC5 non-tumor human cells, and had previously been shown not to affect their growth [4]. In the present work, the *in vitro* cell growth inhibitory effect of this series of compounds was screened in three human tumor cell lines representative of solid tumors. For that, the GI_50_ concentrations were determined for the 27 thioxanthones in MCF-7 (breast adenocarcinoma), NCI-H460 (non-small cell lung cancer, NSCLC) and A375-C5 (melanoma) cells, using the sulforhodamine B assay which allows to indirectly assess cell number by measuring the amount of proteins in cells following treatment [8] (Table 1).

**Table 1:** GI_50_ values determined for the 27 thioxanthones following continuous treatment of the three human tumor cells lines during 48 h. 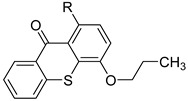

Thioxanthone Number	R	GI_50_ (µM)		
		MCF-7	NCI-H460	A375-C5
**1**	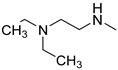	6.1 ± 0.4	6.0 ± 0.3	3.6 ± 1.2
**2**	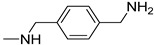	>150	>150	>150
**3**	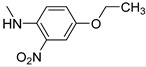	>150	>150	>150
**4**	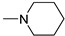	15.0 ± 1.0	13.3 ± 0.7	13.3 ± 0.3
**5**	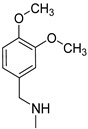	>150	>150	>150
**6**	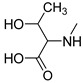	111 ± 11.7	73.0 ± 6.6	73.0 ± 5.5
**7**	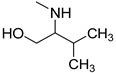	33.0 ± 2.3	27.3 ± 2.7	33.0 ± 2.7
**8**	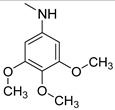	>150	>150	>150
**9**	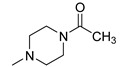	92.7 ± 2.6	89.7 ± 2.4	60.3 ± 1.8
**10**	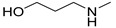	>150	>150	>150
**11**	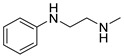	85.0 ± 1	58.3 ± 7.2	81.7 ± 2.9
**12**	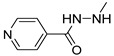	48.0 ± 3.1	38.7 ± 4.4	27.3 ± 5.3
**13**	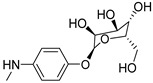	>150	>150	>150
**14**	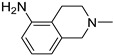	34.0 ± 3.1	39.3 ± 6.4	24.3 ± 3.3
**15**	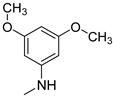	65.7 ± 1.5	59.3 ± 7.9	55.0 ± 1.5
**16**	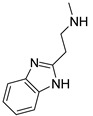	28.3 ± 2.9	22.7 ± 2.5	20.0 ± 2.1
**17**	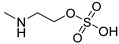	>150	>150	>150
**18**	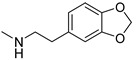	>150	>150	>150
**19**	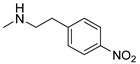	70.8 ± 6.9	61.3 ± 4.1	54.8 ± 9.4
**20**	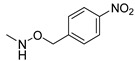	34.3 ± 3.0	35.0 ± 5.2	34.0 ± 4.0
**21**	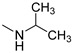	>150	>150	>150
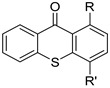				
**22**	R = OCH_3_, R’ = OH	>150	>150	>150
**23**	R=OCH_3_, R’ = OCH_2_CH_2_CH_3_	>150	>150	>150
**24**	R = Cl, R’ = OH	23.3 ± 1.5	21 ± 0.5	15.3 ± 0.9
**25**	R = Cl, R’ = OAc	9.4 ± 0.2	8.7 ± 0.2	8.2 ± 0.3
**26**	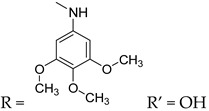	24.3 ± 2.9	23.0 ± 2.5	34.3 ± 1.9
**27**	R = H, R’ = OH	39.0 ± 3.6	28.7 ± 2.2	34.0 ± 6.5

The values presented refer to the mean ± SEM of at least three independent experiments. The maximum vehicle (DMSO) concentration used was 0.25% and was shown not to interfere with cell growth (data not shown). Doxorubicin was used as a positive control (GI_50_ concentrations: 65 ± 8.5 nM in MCF-7, 64 ± 6.8 nM in NCI-H460 and 145 ± 9.8 nM in A375-C5 cells).

Results obtained 48 h after continuous treatment showed that, from all the studied thioxanthones, two compounds, **1** and **25**, were found to be the most potent on the studied cell lines (presenting GI_50_ concentration lower than 10 µM). By comparing the GI_50_ concentration of the compounds in the studied tumor cell lines (derived from solid tumors) with our previous published work on the chronic myeloid leukemia cell line (K562) [4], compound **25** was found to be a more potent growth inhibitory agent towards the solid tumor cell lines (MCF-7, NCI-H460 and A375-C5) than towards the K562 chronic myeloid leukemia cell line (previously determined GI_50_ = 13.57 ± 2.96 µM) [4]. On the other hand, compounds **4**, **12**, **14**, **23** and **26** presented much higher GI_50_ concentrations in the cell lines studied in the present study, when compared with the previously GI_50_ concentrations determined in K562 cells (3.73 ± 1.47 µM, 4.81 ± 4.21 µM, 3.00 ± 0.48 µM, 4.47 ± 1.93 µM, 4.38 ± 0.44 µM, respectively) [4].

The most potent compound **1**, herein designated as the hit compound **TXA1**, was selected to be further studied in order to investigate its cellular mechanism(s) of action. In a previous study, the compound had been found to be non-toxic to non-tumor cells [4]. Interestingly, the amine side chain of compound **1** was structurally very similar to lucanthone, the first thioxanthone described as a potential antitumor agent (originally used as an anti-schistosomal drug) which has already reached clinical trials [2,3].

### 2.2. Synthesis of ***TXA1·HCl***

**Scheme 1:**
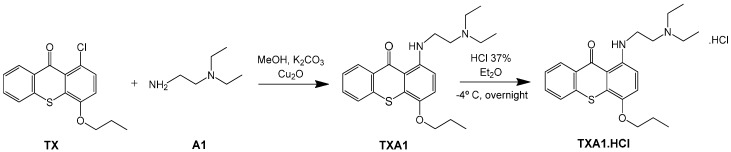
Synthesis of **TXA1** and its hydrochloride salt, **TXA1·HCl**.

Before proceeding with the study of the mechanism of action and in order to improve the solubility and bioavailability characteristic of **TXA1**, its hydrochloride salt (**TXA1·HCl**) was synthesized. As previously published, **TXA1** had been obtained with a 30% yield by an aromatic nucleophilic substitution reaction under copper catalysis [4]. Herein, using a multimilligram approach in a basic media (K_2_CO_3_) in closed vessel, followed by the neutralization reaction of the organic free amine with hydrochloric acid, **TXA1·HCl** was obtained in a 50% overall yield (Scheme 1). This procedure, besides being successful in the formation of the hydrochloride salt of **TXA1**, also allowed its purification without applying chromatographic methodologies.

### 2.3. ***TXA1*** and ***TXA1·HCl*** Inhibit Cell Growth of a Larger Panel of Human Tumor Cell Lines Derived from Solid Tumors

The effect of **TXA1·HCl** was then analyzed in a larger panel of human tumor cell lines, in parallel with **TXA1**. Determination of the GI_50_ concentration of both **TXA1** and **TXA1·HCl** in this larger panel (which also included the previously studied cell lines, see Table 1), allowed to conclude that the hydrochloride form of the compound (**TXA1·HCl**) presented similar activity to the non-soluble **TXA1** form (Table 2). The major difference observed was in the AGS gastric cancer cell line, in which **TXA1·HCl** (GI_50_ = 9.7 µM) was nearly four times more potent than **TXA1** (GI_50_ = 35.3 µM).

To further confirm the cytotoxic effect of **TXA1** and **TXA1·HCl** in these cells, another assay, the MTT assay which measures metabolic activity, was carried out in four of the studied cell lines (A375-C5, MCF-7, NCI-H460 and HeLa).

**Figure 1:**
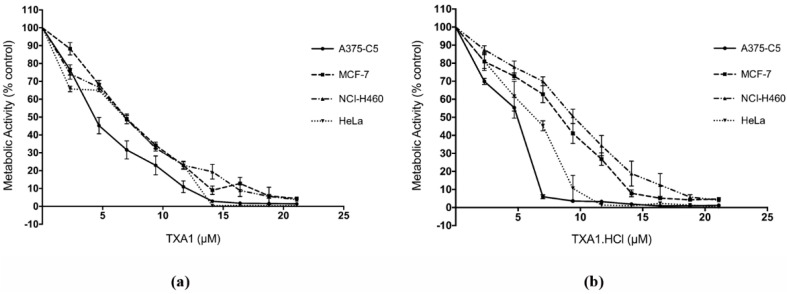
Effect of **TXA1** (**a**) and **TXA1·HCl** (**b**) in four human tumor cell lines, analyzed with the MTT assay. Cells (A375-C5, MCF-7, NCI-H460 and HeLa) were continuously treated with **TXA1** or **TXA1·HCl** for 48 h. Metabolic activity is expressed as percentage of blank (cells treated with medium only). Results are expressed as mean ± SEM of at least three independent experiments. The maximum vehicle (DMSO for **TXA1** and H_2_O for **TXA1·HCl**) concentration was used as control and did not interfere with the cellular metabolic activity (data not shown).

As expected, a dose-dependent decrease in metabolic activity occurred in all of the studied cell lines following continuous cellular treatment with **TXA1** or **TXA1·HCl** (Figure 1). These results were in agreement with the dose-response curves obtained with the SRB assay (data not shown). Moreover, the IC_50_ concentrations (50% inhibitory concentration) determined with the MTT assay for **TXA1** (5.92 ± 0.51 µM in A375-C5, 8.24 ± 0.16 µM in MCF-7, 7.88 ± 0.27 µM in NCI-H460 and 7.10 ± 0.06 µM in HeLa) and for **TXA1·HCl** (4.53 ± 0.24 µM in A375-C5, 8.64 ± 0.42 µM in MCF-7, 9.79 ± 0.50 µM in NCI-H460 and 6.42 ± 0.33 µM in HeLa) were within the same range of the GI_50_ concentrations previously determined with the SRB assay (Table 2).

**Table 2:** GI_50_ values determined for the **TXA1** and **TXA1·HCl** in various tumor cell lines derived from solid tumors.

Compound	GI_50_ (µM)						
	MCF-7	NCI-H460	A375-C5	HeLa	786-O	Caki-2	AGS
TXA1	6.1 ± 0.4 #	6.0 ± 0.3 #	3.6 ± 1.2 #	6.8 ± 0.5	8.1 ± 1.8	11.5 ± 1.8	35.3 ± 2.3
TXA1·HCl	7.8 ± 0.9	6.9 ± 1.3	3.8 ± 0.8	6.4 ± 0.6	9.0 ± 1.1	9.3 ± 0.8	9.7 ± 0.2

Values presented refer to the mean ± SEM of at least three independent experiments. The maximum vehicles (DMSO for **TXA1** and H_2_O for **TXA1·HCl**) concentration used was 0.25% and did not interfere with cell growth (data not shown). Doxorubicin was used as a positive control [GI_50_ concentrations: 65 ± 8.5 nM in MCF-7, 64 ± 6.8 nM in NCI-H460, 145 ± 9.8 nM in A375-C5, 20.7 ± 0.8 nM in HeLa, 135 ± 6.2 nM in 786-O, 152.3 nM in Caki-2 (from two experiments only), 90 ± 5 nM in AGS]. # data also included in Table 1 as the GI_50_ concentration of compound **1**.

### 2.4. The Effect of ***TXA1·HCl*** in MCF-7 Cells is Irreversible

**Figure 2:**
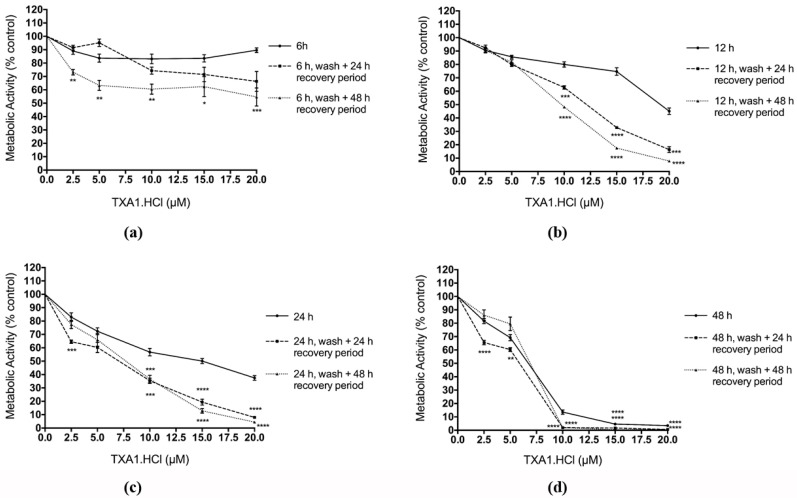
Analysis of the reversibility of the effect of **TXA1·HCl** in MCF-7 cells, analyzed with the MTT assay. Cells were treated with increasing concentrations of **TXA1·HCl** for 6 h (**a**); 12 h (**b**); 24 h (**c**) or 48 h (**d**) and then incubated for an additional 24 or 48 h in compound-free medium. Metabolic activity is expressed as percentage of blank (cells treated with medium only). Results are expressed as mean ± SEM from three independent experiments. * *p* ≤ 0.05; ** *p* ≤ 0.01; *** *p* ≤ 0.001; **** *p* ≤ 0.0001 when comparing treatment *vs.* control (H_2_O).

In order to investigate if the cytotoxic effects of **TXA1·HCl** were irreversible, MCF-7 cells were treated with increasing concentrations of this compound for 6, 12, 24 or 48 h, after which the compound was removed and cells left to recover in fresh medium for an additional 24 or 48 h. The reason to include the 6 h treatment was that preliminary experiments showed that a 10 h incubation period with the compound was enough to induce morphology changes in MCF-7 cells (data not shown). Following the recovery period, the MTT assay was performed and corresponding dose-response curves were generated (Figure 2). Results showed that the effect of **TXA1·HCl** was both time- and dose-dependent. Although no changes were observed in the cellular metabolic activity (measured with MTT assay) following 6 h of treatment with **TXA1·HCl**, a significant decrease in metabolic activity was observed in these cells upon 48 h of recovery. This seems to indicate that 6 h treatment with **TXA1·HCl** is sufficient to interfere with the mechanisms involved in cell survival and proliferation, even though this effect can only be observed latter. In addition, **TXA1·HCl** treatment for 12 h resulted in a pronounced decrease in cellular metabolic activity (when comparing with the 6 h exposure). Moreover, no recovery was observed upon the 24 or the 48 h recovery period, when an even higher decrease in the cellular metabolic activity was observed. The same trend was observed in cells initially treated for 24 or 48 h with **TXA1·HCl**, although the reduction in cellular metabolic activity was increasingly higher in these situations. These results indicate that the **TXA1·HCl** effect on MCF-7 cells is irreversible.

**Figure 3:**
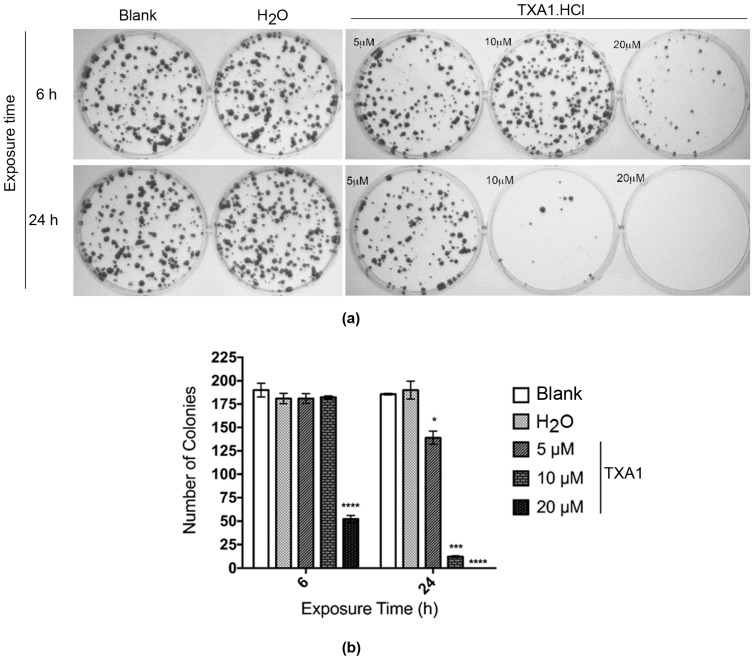
Effect of **TXA1·HCl** on the colony forming efficiency of MCF-7 cells. Cells were treated for 6 or 24 h with 5, 10 and 20 μM **TXA1·HCl**. Results were analyzed 14 days after recovery of the cells previously treated with **TXA1·HCl**. Representative images (**a**) and analysis of the number of colonies (**b**). Results are expressed as mean ± SEM, from three independent experiments. * *p* ≤ 0.05; *** *p* ≤ 0.001; **** *p* ≤ 0.0001 when comparing treatment *vs.* control (H_2_O).

In order to confirm the irreversibility effect of **TXA1·HCl**, the colony forming (or clonogenic) assay was carried out in the MCF-7 cells. For this, cells were treated with 5, 10 and 20 μM **TXA1·HCl** for 6 and 24 h, after which the compound was removed and the cells allowed to recover for 14 days. The number of colonies formed was then determined (Figure 3).

As expected, **TXA1·HCl** caused a time- and concentration-dependent decrease in the colony-forming capability of MCF-7 cells. Treatment with 20 μM **TXA1·HCl** for 6 h only resulted in a significant reduction of approximately 70% in the number of colonies when compared with the treatment with solvent control. The reduction was even more evident when the cells were treated for 24 h with the same concentration (20 μM), with no colonies having been observed. In addition, 24 h treatment with lower concentrations (5 and 10 μM) significantly decreased the colony forming capability of cells approximately by 30% and 95%, respectively.

Naturally-occurring xanthones have shown similar effects in tumor cells. For example, α-mangostin, a prenylated xanthone isolated from the mangosteen fruit, inhibited colony formation in prostate cancer cells [9]. Also, the caged xanthone gambogic acid significantly inhibited the colony formation capability of glioblastoma cells [10].

Overall, the results obtained from the MTT assay and from the colony forming assay demonstrate that, as for other xanthones, **TXA1·HCl** may act as a cytotoxic, rather than as a cytostatic compound, with its effect being irreversible following at least 6 h exposure. Therefore, this compound is possibly an inducer of cell death.

### 2.5. ***TXA1·HCl*** Induces Cell Death in MCF-7 Cells

Several studies have shown that one of the main mechanisms involved in the antitumor effect of xanthones is the induction of cell death by apoptosis. For example, γ-mangostin was shown to induce apoptosis in human colon cancer cells [11]. In addition, *α*-mangostin induced cell cycle arrest and apoptosis in breast cancer [12] and in prostate cancer [9] cells. Also, gambogic acid induced cell cycle arrest and apoptosis in glioblastoma cells [10]. Pyranocycloartobiloxanthone A, a xanthone isolated from *Artocarpus obtusus*, induced apoptosis in MCF-7 cells [13] and a xanthone isolated from *Garcinia oblongifolia* induced apoptosis in hepatocellular carcinoma cells [14].

Preliminary experiments, in which DAPI-stained DNA of **TXA1·HCl** treated cells was observed by fluorescence microscopy, showed the presence of bright DAPI-stained micronuclei, suggestive of cell death by apoptosis (data not shown).

**Figure 4:**
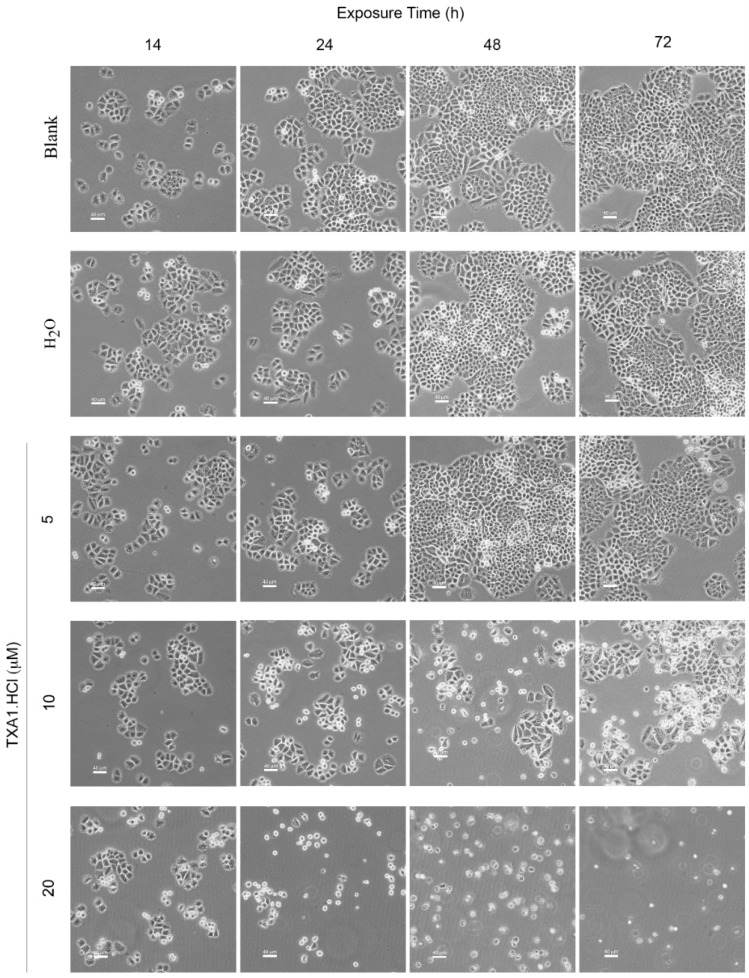
Effect of **TXA1·HCl** treatment in MCF-7 cells, analyzed by phase contrast microscopy. Cells were treated for 14, 24, 48 and 72 h with medium only (Blank), H_2_O and **TXA1·HCl** (5, 10 and 20 µM). Images are representative of at least three independent experiments. Scale bar = 40 μm.

Therefore, the effects of **TXA1·HCl** in MCF-7 cellular morphology was further studied. A time and dose-dependent effect on cellular morphology was clearly observed (Figure 4), such as cell shrinkage, detachment and membrane blebbing, which were suggestive of apoptosis [15]. These morphological alterations were observed with the 10 μM and higher **TXA1·HCl** concentrations. The same was observed in HeLa cells following treatment with this compound (Supplementary Figure S1). Furthermore, regular monitoring of the cells revealed that these effects were already evident following 10–12 h treatment (data not shown). To further confirm the effect of **TXA1·HCl** on cell death, the TUNEL assay was carried out.

**Figure 5:**
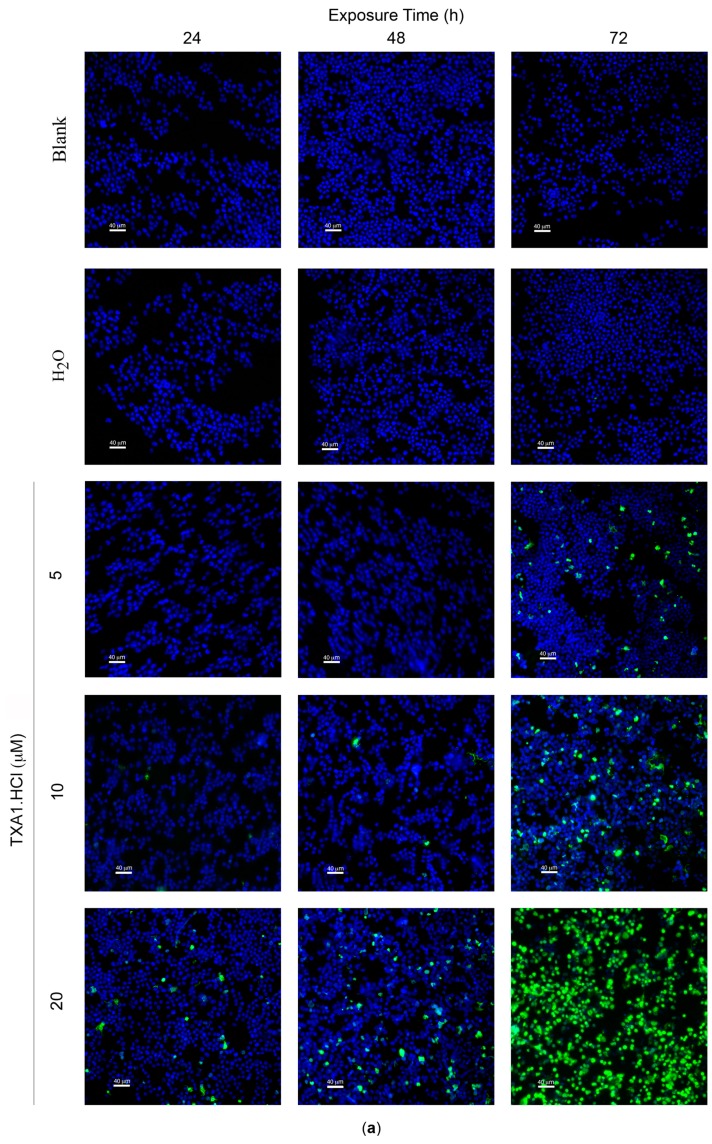

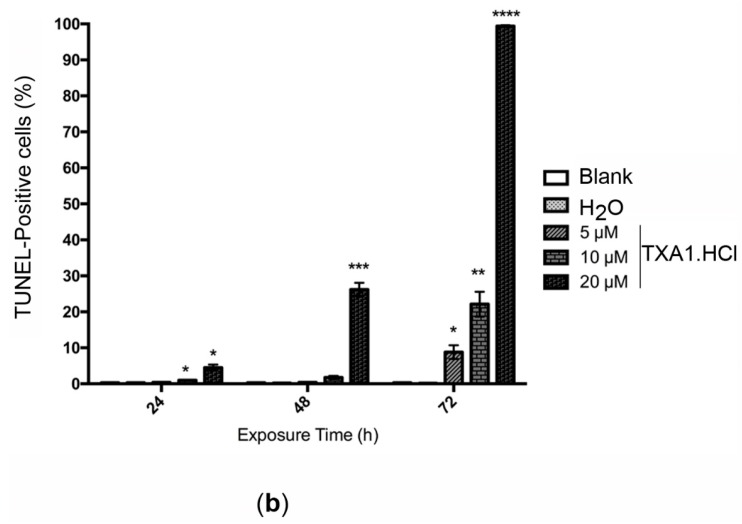
Effect of **TXA1·HCl** treatment in MCF-7cell death, analyzed with the TUNEL assay. Cells were treated for 24, 48 and 72 h with medium only (Blank), H_2_O and **TXA1·HCl** (5, 10 and 20 µM). (**a**) Representative fluorescence microscopy images showing increasing TUNEL-positive cells (green) as induced by 5, 10 or 20 µM of **TXA1·HCl**, while few or no TUNEL-positive cells were observed in control cultures (Blank and H_2_O). DNA was stained with DAPI (blue); bar = 40 μm; (**b**) Quantification of the levels of cell death. Results are the mean ± SEM from at least three independent experiments. * *p* ≤ 0.05; ** *p* ≤ 0.01; *** *p* ≤ 0.001; **** *p* ≤ 0.0001 when comparing treatment *vs.* control (H_2_O).

Results showed a time- and dose-dependent increase in the percentage of TUNEL-positive cells (Figure 5), suggestive of increased apoptosis. While no induction of cell death was observed with the lower concentration of **TXA1·HCl** (5 µM) at the earlier time points (24 and 48 h), the highest treatment concentration (20 µM) resulted in increased levels of TUNEL-positive cells at these time points. When analyzing the effect of a longer period of treatment (72 h), a clear and significant increase in the percentage of cell death was observed following treatment with 5 μM (8.8% ± 1.9%), 10 μM (22.1% ± 3.4%) or 20 μM (nearly all cells were dead).

To further confirm that apoptosis was the cause of this cell death, flow cytometry analysis following Annexin V-FITC and propidium iodide (PI) staining was carried out. This is a specific apoptosis assay (detecting both early and late stage apoptosis) and also more sensitive that the TUNEL assay [16,17]. Results (Table 3) indicated that 48 h treatment with 5 μM **TXA1·HCl** did not induce apoptosis, in agreement with the above TUNEL assay results. However, treatment with 10 μM **TXA1·HCl** induced apoptosis in approximately 50% of the cells analyzed. This percentage of apoptosis was very high when compared to that of TUNEL-positive cells determined for the same concentration and at the same time-point (1.8% ± 0.8%). Nevertheless, as previously mentioned, this is because the Annexin-V FITC/PI analysis is much more sensitive than the TUNEL assay, allowing to detect apoptosis at earlier stages (early apoptosis) whereas the TUNEL assay only detects cells with breaks on the DNA (late apoptosis) [18].

**Table 3:** Levels of apoptosis in MCF-7 cells following treatment with **TXA1·HCl**.

Treatment		Apoptotic Cells (%)
Blank		11.0 ± 0.6
H_2_O		10.1 ± 1.4
TXA1·HCl	5 μM	13.7 ± 0.4
	10 μM	51.5 ± 5.0 *

Cells were treated for 48 h with medium (Blank), H_2_O or with **TXA1·HCl** (5 µM or 10 µM). Results refer to the values determined by flow cytometry analysis following Annexin V-FITC/PI staining and are the mean ± SEM of at least three independent experiments. * *p* ≤ 0.05 when comparing treatment *vs.* control (H_2_O).

This further supports the results previously obtained, in which only the higher concentrations of **TXA1·HCl** (or prolonged drug treatment) induced cell death.

## 3. Experimental Section

### 3.1. General Information

Purification of compounds was performed by flash chromatography using silica gel 60 (0.040–0.063 mm, Merck, Darmstadt, Germany) and chromatography flash cartridges (GraceResolv, Grace Company, Deerfield, IL, USA). Thin layer chromatography (TLC) using Merck silica gel 60 (GF254) was used for monitoring reactions and as purity criteria. The reactions were considered finished, when one of the reagents was no longer detectable by TLC or remained constant over 30 min. The purity of **TXA1·HCl** was >95% determined by HPLC-DAD analysis using an isocratic elution of MeOH:H_2_O 8:2 nade alkaline with TEA (1%) at a constant flow rate of 1.0 mL·min^−1^ [4]. Melting points were obtained in a Köfler microscope. IR spectra were measured on an Mattson Genesis series FTIR (ATI, Unicam Sistemas analíticos, Linda-a-Velha, Portugal; software: WinFirst v.2.10) spectrophotometer in KBr microplates (cm^−1^). ^1^H spectra were taken in DMSO-*d*_6_ DRX-500 (500.13 MHz for ^1^H) spectrometer (Bruker, Biosciences Corporation, Billerica, MA, USA). Chemical shifts are expressed in δ_H_ (ppm) values relative to Me_4_Si as an internal reference. **TX** and **A1** were obtained from Sigma-Aldrich (Steinhein, Germany) and were of *pro analysis* grade.

### 3.2. Compound Synthesis

Synthesis of compounds **1**–**27** was performed according to the described procedure [4]. The purity of each compound was determined by HPLC–DAD analysis using an isocratic elution of MeOH:H*_2_*O basified with TEA (1%) or acidified with CH*_3_*COOH (1%) at a constant flow rate of 1.0 mL/min [4]. All tested compounds possessed a purity of at least 95%. Compounds were dissolved in DMSO.

### 3.3. Multimilligram Synthesis of ***TXA1***

Four individual mixtures of **TX** (500 mg; 2.0 mmol), amine **A1** (0.44 mL, 4.0 mmol), K_2_CO_3_ (442 mg; 4.0 mmol), Cu_2_O (2 mg) and MeOH (20 mL) were placed in closed vessels. The mixtures were allowed to react in parallel at 100 °C, with no stirring, during 24 h. The reaction mixtures were recombined, filtered and concentrated under reduced pressure furnishing a dark brown crude product. The crude product was dissolved with diethyl ether (30 mL), made alkaline with an aqueous solution of NaOH 20% and washed with water (3 × 30 mL). The ether fraction was dried (Na_2_SO_4_), filtered and concentrated under vacuum. This concentrate was used to the next reaction without further purification.

### 3.4. Synthesis of ***TXA1*** Hydrochloride *(**TXA1·HCl**)*

The ether solution obtained in the previous section was cooled at −4 °C and a concentrated hydrochloric solution 37% (1 mL) was added. An orange precipitate was formed and it was placed at −4 °C overnight. The solid thus obtained was filtered, washed with 90 mL of diethyl ether, and dried in a desiccators containing phosphorus pentoxide furnishing a dark orange solid corresponding to **TXA1·HCl** (1.39 g, 51%). m.p. >48 °C dec; IR (KBr) υ_max_: 3425; 2960; 2930; 2874; 2655; 1614; 1573; 1508 cm^−1^; ^1^H-NMR (DMSO-*d*_6_, 500.13 MHz) δ_H_: 7.52 (1H, s), 6.71 (1H, s), 6.61 (1H, s), 6.33 (1H, s), 6.32 (1H, s), 5.90 (1H, s), 3.19 (4H, m), 3.03 (6H, m), 1.19 (2H, m), 1.07 (6H, t, *J* = 7.1), 0.56 (3H, t, *J* = 7.1).

### 3.5. Cell Culture

A375-C5 (malignant melanoma), MCF-7 (breast adenocarcinoma), NCI-H460 (non-small cell lung cancer), 786-O (renal cell adenocarcinoma) and AGS (gastric adenocarcinoma) cell lines were cultured in RPMI-1640 medium with UltraGlutamine I and 25 mM Hepes (Lonza, Basel, Switzerland) supplemented with 5% Fetal bovine serum (FBS, Biowest, Nuaillé, France). Caki-2 (clear cell carcinoma) cells were cultured in McCoy’s 5A (Life Technologies, Carlsbad, CA, USA) supplemented with 10% (*v*/*v*) FBS. HeLa (cervical adenocarcinoma) cells were cultured in DMEM (PAA Laboratories, Pasching, Austria) supplemented with 10% (*v*/*v*) FBS. All cell lines were grown in monolayer cultures and kept permanently in exponential growth in a humidified incubator, at 37 °C and a 5% CO_2_ atmosphere. Cell number and viability were assessed with Trypan blue exclusion assay.

### 3.6. In Vitro Screening for Tumor Cell Growth Inhibition with the Sulforhodamine B Assay

The sulforhodamine B (SRB) assay was carried out according to the procedure adopted by the NCI’s Developmental Therapeutics Program [8,19,20]. Briefly, cells were plated in 96-well plates (5 × 10^3^ cells/well for MCF-7 and NCI-H460 cells; 7.5 × 10^3^ cells/well for A375-C5; 2 × 10^3^ cells/well for HeLa cells) in the respective culture medium and incubated at 37 °C for 24 h. Cells were then incubated for 48 h with 5 serial dilutions of the each compound (at a maximum of 37.5 µM for **TXA1**,15 µM for **TXA1·HCl** and 150 µM for all the other compounds) at 37 °C and 5% CO_2_. Cells were with 10% (*w*/*v*) trichloroacetic acid (TCA), washed with distilled water, and proteins stained with 0.4% (*w*/*v*) SRB. After washing with 1% (*v*/*v*) acetic acid, bound SRB was solubilized with 10 mM Tris base and absorbance measured at 510 nm. For each compound analyzed, the GI_50_ (the concentration that inhibits 50% of cell growth) was determined by interpolation of the dose-response curve graphic obtained using a NCI datasheet.

### 3.7. Confirmation of the Activity of the Hit Compounds *(**TXA1*** and ***TXA·HCl**)* with the 3-(4,5-Dimethyl-thiazolyl-2)-2,5-diphenyltetrazolium Bromide (MTT) Assay

Cells were plated in 96-well plates (2.0 × 10^3^ cells/well for A375-C5 cells, 5 × 10^3^ cells/well for MCF-7 and NCI-H460 cells and 5 × 10^3^ cells/well for HeLa cells) in the respective culture medium and incubated at 37 °C for 24 h. Cells were then incubated for 48 h with **TXA1** or **TXA1·HCl** (concentrations ranging from 2.3–21.1 μM) at 37 °C and 5% CO_2_. MTT, previously dissolved in PBS, was then added to each well (0.5 mg/mL final concentration) and incubated for 4 additional hours in the same conditions. Formazan crystals were then solubilized by adding 100 μL solubilization solution. Absorbance values were read at 550 nm. Dose-response curves were plotted and IC_50_ values were obtained using the GraphPad Prism^®^ version 6.0c software.

### 3.8. Reversibility Analysis of the Effect of the Hit Compound, with the MTT Assay and the Colony Forming Assay

For the reversibility studies, the MTT was carried out (as described in the previous section) in MCF-7 cells after treatment for 6, 12, 24 or 48 h with 5, 10 or 20 µM **TXA1·HCl**. In addition, after each treatment period, **TXA1·HCl** was removed, cells were washed with PBS and further incubated for additional 24 and 48 h in compound-free cell culture medium at 37 °C and 5% CO_2_. MTT assay was also carried out at the end of these experiments.

For the colony forming assay, MCF-7 cells (7.5 × 10^2^ cells/well in 6-well plates) were plated in cell culture medium and incubated for 24 h at 37 °C in 5% CO_2_ to allow cell adhesion. Cells were then treated for 6 h or 24 h with 5, 10 or 20 µM **TXA1·HCl**. Cells were also incubated with medium only (Blank) or with the corresponding concentrations of H_2_O (Control). After treatment, the compound-containing medium was discarded and cells further incubated with fresh cell culture medium for 14 days. The resulting colonies were fixed with 3.7% (*v*/*v*) paraformaldehyde and stained with 0.05% (*w*/*v*) crystal violet (in distilled water). All plates were photographed in a Gel Doc™ XR system (Bio-Rad, Hercules, CA, USA) and colonies were manually counted.

### 3.9. Study of the Effect of the Hit Compound on Cell Death

MCF-7 (1 × 10^5^ cells/well) were plated in 6-well plates in cell culture medium and incubated for 24 h at 37 °C in 5% CO_2_ to allow cell adhesion. Cells were then treated for 24, 48 and 72 h with 5, 10 or 20 µM **TXA1·HCl**. Cells were also incubated with medium only (Blank) or with the higher volume of the solvent (H_2_O, Control).

For morphology analysis, cells were observed under phase contrast microscopy, in a Nikon Eclipse TE2000-U microscope equipped with a DXM1200F digital camera and controlled by Nikon ACT-1 software (Melville, NY, USA), and images were acquired with a 40× magnification.

For the TUNEL assay, the DeadEnd™ Fluorometric TUNEL System (Promega, Madison, WI, USA) was used, according to the manufacturer’s instructions. Briefly, after cytospins were prepared, cells were fixed in 4% (*v*/*v*) paraformaldehyde in PBS, permeabilized with 0.2% (*v*/*v*) Triton X-100 in PBS and incubated with TUNEL reaction mixture (enzyme dilution 1:20). Slides were then mounted with Vectashield with DAPI and analyzed by fluorescence microscopy under a Zeiss Spinning Disc AxioObserver Z.1 SD microscope, coupled to an AxioCam MR3 camera (Carl Zeiss, Jena, Germany). The percentage of TUNEL positive labelled cells was determined by a counting at least 1000 cells for each condition [21].

For flow cytometric analysis of apoptotic cell death, the “Annexin V-FITC Apoptosis Detection kit” (Bender MedSystems, Vienna, Austria) was used as previously described [22]. Briefly, cell pellets were resuspended in binding buffer and incubated with Annexin V-FITC and with propidium iodide. Cells were then analyzed by flow cytometry in a BD Accuri C6 cytometer (BD Biosciences, Erembodegem, Belgium). All data was analyzed using the FlowJo 7.6.5 software (Tree Star, Inc., Ashland, OR, USA), plotting at least 20,000 events per sample.

### 3.10. Statistical Analysis

All presented data were obtained from at least three independent. All data was statistically analyzed with the two-tailed paired Student’s *t*-test. Results were considered statistically significant when *p* ≤ 0.05.

## 4. Conclusions

In the present study, the tumor cell growth inhibitory effect of a series of 27 thioxanthones was analyzed in three human tumor cell lines derived from solid tumors: MCF-7 (breast adenocarcinoma), NCI-H460 (non-small cell lung cancer) and A375-C5 (melanoma). This revealed the compound 1-{[2-(diethylamino)ethyl]amino-4-propoxy-9*H*-thioxanthen-9-one (**TXA1**) as the most potent compound from this small library. Its hydrochloride derivative, **TXA1·HCl**, was synthesized in order to improve the solubility as well as the bioavailability of this compound. Both **TXA1** and **TXA1·HCl** decreased MCF-7 tumor cell growth. The effect of **TXA1·HCl** was found to be irreversible. Additionally, the effect of this hit compound was found to be, at least in part, due to cell death by apoptosis. Future work will allow to further elucidate its mechanisms of action.

## Supplementary Materials

Supplementary materials can be accessed at: http://www.mdpi.com/1420-3049/21/1/81/s1.
