# Supplementary material for: Screening a Small Library of Xanthones for Antitumor Activity and Identification of a Hit Compound which Induces Apoptosis
Source: Molecules. 2016 Jan 13;21(1):81. doi: 10.3390/molecules21010081 (PMC6274047; doi:10.3390/molecules21010081)
Supplement: Supplementary file 1 [file molecules-21-00081-s001.pdf]

Supplementary Materials: Screening a Small Library of Xanthones for Antitumor Activity and Identification of a Hit Compound which Induces Apoptosis

João Barbosa, Raquel T. Lima, Diana Sousa, Ana Sara Gomes, Andreia Palmeira, Hugo Seca, Kantima Choosang, Pannee Pakkong, Hassan Bousbaa, Madalena M. Pinto, Emília Sousa, M. Helena Vasconcelos and Madalena Pedro

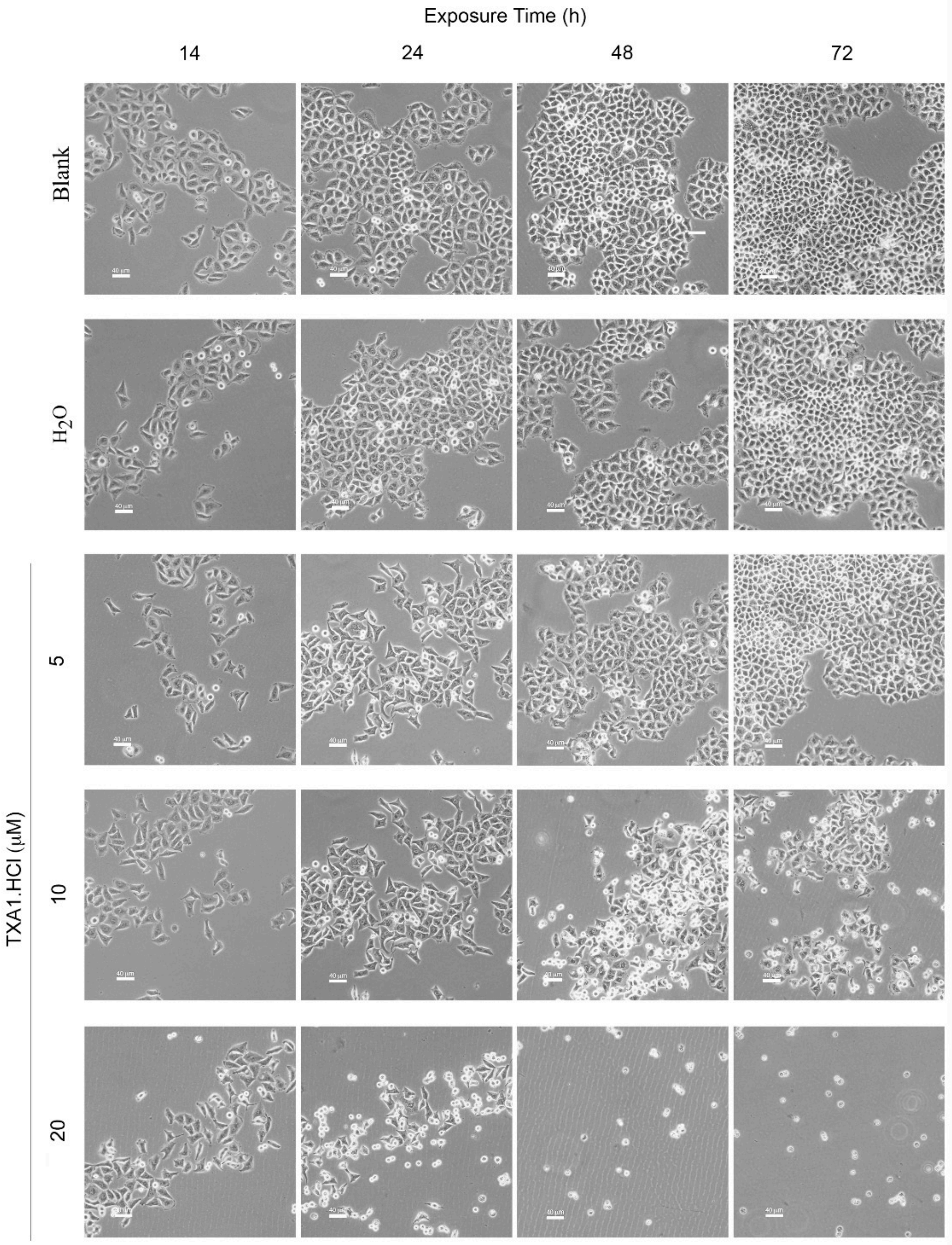

Figure S1. Effect of TXA1.HCl treatment in HeLa cells, analyzed by phase contrast microscopy. Cells were treated for 14, 24, 48 and 72 h with medium only (Blank), H2O and TXA1·HCl (5, 10 and 20 μM). Images are representative of at least 3 independent experiments. Scale bar = 40 μm.
